# Facile synthesis of nitrogen-doped graphene quantum dots as nanocarbon emitters for sensitive detection of catechol†

**DOI:** 10.1039/d2ra04209f

**Published:** 2022-09-09

**Authors:** Xiayi Liang, Wenhao Zhang, Mengqi Zhang, Guanhua Qiu, Yuhong Zhang, Tao Luo, Cunqing Kong

**Affiliations:** Guangxi Medical University Cancer Hospital, Guangxi Medical University 71 Hedi Road Nanning 530021 China cunqing_k@163.com; The First Affiliated Hospital of Guangxi Medical University, Guangxi Province Nanning 530021 China

## Abstract

Development of nanomaterial-based electrochemiluminescence (ECL) emitters is highly desirable for the fabrication and wide applications of ECL sensors. Herein, nitrogen-doped graphene quantum dots (NGQDs) were easily synthesized as nanocarbon emitters with anodic ECL for sensitive ECL determination of catechol (CC). Facile synthesis of NGQDs was easily achieved using molecular fusion of a carbon precursor in a one-step hydrothermal process. The synthesis has advantages of simple and convenient operation and high yield. The as-prepared NGQDs have uniform size, good crystallinity, single-layered graphene structure, and excitation-independent fluorescence. In the presence of hydrogen peroxide (H_2_O_2_), NGQDs exhibit high anodic ECL owing to the presence of functional hydrazide groups. As CC could significantly reduce the ECL intensity of NGQDs, sensitive determination of CC was realized with a linear range from 100 nM to 10 μM and 10 μM to 60 μM with a low limit of detection (LOD, 42 nM). The determination of CC in environmental water was also achieved with high reliability.

## Abstract 2

Nitrogen-doped graphene quantum dots are easily synthesized and can be used as nanocarbon emitters for sensitive electrochemiluminescence detection of catechol.

## Graphical abstract

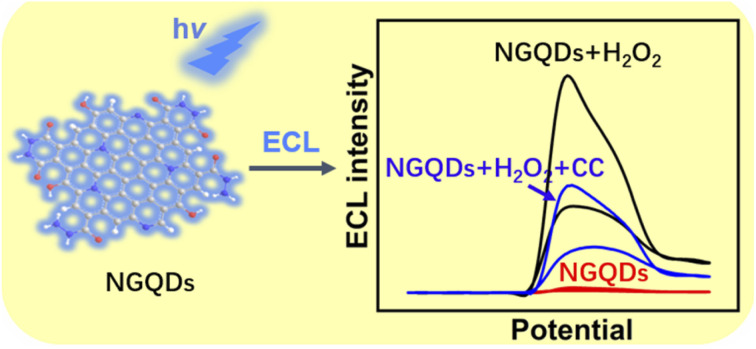

## 1. Introduction

Catechol (CC) is recognized as a carcinogen by the international agency for research on cancer of the World Health Organization.^1–3^ As an important industrial raw material and chemical synthesis intermediate, CC is widely used in coatings, fragrance, resins and other industries.^4–6^ However, conventional water treatment processes cannot effectively remove this substance. Thus, CC is ubiquitous in environmental and drinking water. In addition, CC is not easily naturally degraded. Long-term consumption can damage the central nervous system and liver of the human body, leading to huge environmental and health risks. Therefore, convenient and sensitive determination of CC is of great significance for environmental and health risk assessment.

Until now, strategies that have been reported to detect CC includes chromatography, spectrophotometry, electrochemical methods, chemiluminescence, *etc.*^7–11^ Among them, electrochemiluminescence (ECL) has attracted great research interest in recent years as an chemiluminescence generated in the process of electrochemical scanning, which produces special electro-generated substances and then forms an ex-cited state through electron transfer reaction to emit light.^10–12^ Compared with other luminescence strategies (*e.g.* fluorescence), ECL does not need an external light source, which reduces the background interference caused by light scattering or spontaneous fluorescence.^13–16^ In comparison with electrochemical detection, ECL also possesses high sensitivity and potential for visual detection and imaging analysis.^17–21^ Thus, ECL has unique advantages including fast response, high sensitivity, wide linear range, good controllability, and simple instrument. ECL emitter is of great significance to improve the performance of the ECL sensing system. In addition to molecular luminescent probes (such as ruthenium complexes), nanomaterial-based ECL emitters have attracted much attention in recent years owing to their different sizes, morphologies, and chemical compositions.^15,22–24^ In addition, it has been proven that nanoemitters can be confined in porous nanomaterials, solid film or gel, demonstrating great potential in solid-state luminescence sensing, visual detection, *etc.* Thus, development of novel nanoemitters and their application in environmental analysis is crucial.

Quantum dots (QDs) are attractive because of their excellent electrochemical and optical properties.^24–27^ Amongst, graphene quantum dots (GQDs) are the new member of nanocarbon family. As small-sized graphene fragment or zero-dimensional (0D) graphene with the lamellar structure of graphene and small size (usually less than 10 nm), GQDs show a series of excellent characteristics including high specific surface area, excellent water solubility, good stability, and biocompatibility.^28–30^ Owning to the open bandgap resulting from the significant quantum confinement effect, GQDs exhibit excellent fluorescence (photoluminescence) and ECL (electrogenerated chemiluminescence) characteristics,^31–34^ and have great promising in the fields of optoelectronic devices,^28^ biological imaging,^35,36^ photocatalysis,^37^ and (bio)chemical sensors.^38,39^ The strategies for the preparation of GQDs can be divided into top-down and bottom-up methods. The top-down method uses large sheets of graphene oxide or graphene as raw materials, and nano-scale GQDs are formed by oxidative cutting through hydrothermal, solvothermal, or electrochemical methods, *etc.*^40^ However, GQDs synthesized using top-down method suffer from uncontrollable size and low quantum yields. For comparison, the bottom-up method uses small molecular substances as precursors and GQDs are produced through chemical fusion of precursors in microwave-assisted methods, hydrothermal or solvothermal processes, *etc.*^41–44^ GQDs synthesized by this method have advantages of highly controllable morphology and size. Although there have been some reports of GQDs as nano-ECL emitters, most of them are based on cathodic electrochemiluminescence at lower negative potentials. The synthesis and application of GQDs with good ECL performance at low anodic potentials remain challenging. Therefore, development of GQD-based ECL emitter with anodic electrochemiluminescence through simple process and its application in sensitive ECL detection of catechol is highly desirable.

**Fig. 1:**
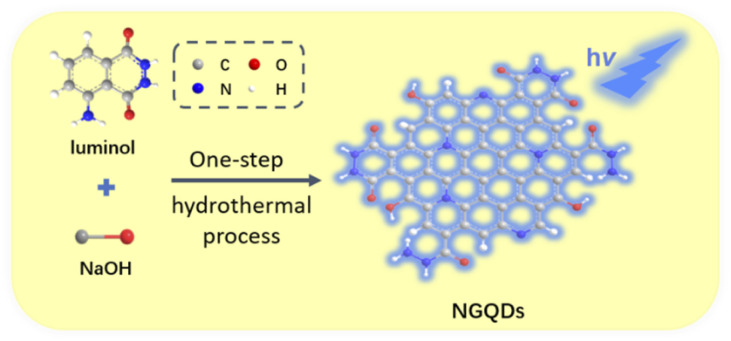
Schematic illustration for one-step preparation of fluorescent NGQDs using hydrothermal process with luminol as precursor.

In this work, we demonstrate an electrochemiluminescence sensor based on nitrogen doped GQDs (NGQDs), which can act as ECL emitter and enable sensitive de-termination of catechol. As shown in Fig. 1, NGQDs are easily synthesized through one-step hydrothermal synthesis using luminol as precursor. The as-prepared NGQDs have uniform size and single-layered graphene structure. In addition, NGQDs have bright blue fluorescence with high absolute quantum yield of (13.2%). When the excitation wavelength changes, the maximum fluorescence emission wavelength of NGQDs does not change, indicating an excitation-independent fluorescence. Due to the presence of hydrazide group, N-GQDs exhibit strong anodic ECL in presence of hydrogen peroxide (H_2_O_2_). Since catechol can inhibit the ECL signal of NGQDs, sensitive detection of catechol can be achieved. Compared with solution-phase ECL luminescent probes, NGQDs promise great potential in optoelectronic devices, composite material, and resonance energy transfer (RET) system because of the possibility of confinement in porous nanomaterials, gel, film *etc.*

## 2. Materials and methods

### 2.1 Chemicals and materials

Luminol and catechol were obtained from Aladdin Chemistry Co. Ltd (Shanghai, China). Na_2_HPO_4_, NaH_2_PO_4_, KCl, H_2_O_2_ (30%) were purchased from Hangzhou Shuanglin Chemical reagent (Hangzhou, China). Glassy carbon electrode (GCE, 3 mm in diameter), Pt wire electrode, and Ag/AgCl electrode were purchased from Shanghai Chenhua Electrochemical Co., Ltd (China). The lake water was collected from the lake in Guangxi Medical University (Nanning, China). All chemicals were of analytical grade and used without further purification. To prepare aqueous solutions, ultrapure water (18.2 MΩ cm) was obtained using Mill-Q system (Millipore, USA) and used throughout the work.

### 2.2 One-step synthesis of NGQDs

NGQDs were synthesized using one-step hydrothermal method. Luminol was used as the precursor and sodium hydroxide was used as the synthesis medium. Briefly, luminol (0.4 mg mL^−1^) was dissolved in sodium hydroxide (0.1 M). After 5 min of ultra-sound treatment (frequency 40 kHz, power 120 W), the solution was transferred to a Teflon-lined autoclave and heated at 180 °C for 10 h. After cooled to room temperature, the obtained solution was filtered using 0.22 μm filter membrane followed by dialysis with a cut-off molecular weight of 1000 Da for 48 h. Then, the final solution without the unreacted small molecules was freeze-dried to obtain NGQD power. The resulting NGQDs could be easily re-dispersed in water.

### 2.3 Experiments and instrumentations

The size and lattice structure of NGQDs were investigated by transmission electron microscopy (TEM). TEM images were acquired on a JEM-2100 transmission electron microscope (JEOL Co., Ltd, Japan) with a working voltage of 100 kV. Before measure-ment, NGQDs were dropped on the ultrathin carbon film and dried naturally. The thickness of NGQDs was characterized by atomic force microscopy (AFM). NGQDs were drop-coated onto freshly exfoliated mica. Then, AFM images were acquired using tapping mode on a Bruker Multimode 8 (Bruker Inc., USA). The ultraviolet-visible (UV-vis) absorption spectrum was recorded using UV-2450 spectrometer (Shimadzu, Japan). Fluorescence measurements including excitation and emission spectra and the absolute fluorescence quantum yield were performed on a FL 3C-11 fluorescence spectrometer (Hariba Scientific, USA). For the measurement of the absolute fluorescence quantum yield, the used excitation and emission wavelengths were 300 nm and 420 nm, respectively. ECL measurements were performed using a CHI 832C electrochemical workstation (CH Instruments, China) and a MPI multifunctional ECL analyzer (Xi'an Remex Analytical Instrument Ltd, China). A convenient three-electrode system was used. Amongst, GCE electrode was used as the working electrode. The reference electrode adopts Ag/AgCl electrode (saturated with KCl solution) and the counter electrode is platinum wire electrode. The voltage of photomultiplier tube (PMT) was set as 600 V. XRD pattern was measured using D8 Advance X-ray diffractometer (Bruker, Germany).

### 2.4 ECL determination of catechol

For the ECL determination of catechol, phosphate buffer solution (PBS, 0.1 M, pH = 9.0) containing KCl (0.1 M), NGQD (100 μg mL^−1^) and H_2_O_2_ (1 mM) was used as the detection medium. Before use, the working electrode, GCE, was polished with 0.5 μm, 0.3 μm and 0.05 μm alumina powder successively. Then, it was ultrasonically cleaned in ethanol and ultrapure water, respectively. When different concentrations of catechol were added, the ECL signals generated during cyclic voltammetry (CV) scan were measured. The scanning potential ranged from −0.2 V to 0.8 V with a scan rate of 0.1 V s^−1^. For real sample analysis, the lake water was firstly filtered using a 0.22 μm film to remove large solids and impurities. The reliability of the ECL determination of CC based on NGQD emitter was evaluated using the standard addition method. An artificial amount of CC was added into the lake water. The obtained samples with added CC were then diluted by a factor of 10 using the detection medium and the ECL intensity was measured.

## 3. Results and discussion

### 3.1 Facile synthesis of NGQDs using one-step hydrothermal process

As a new zero dimensional (0D) nanocarbon material, graphene quantum dots (GQDs) have strong quantum confinement and edge effect because their sizes are smaller than Bohr radius, leading to excellent fluorescence and electrochemiluminescence properties. The mostly reported GQDs with ECL properties are mainly produced by cutting large-sized carbon materials including graphite, graphene oxide, carbon black, carbon nanotubes, *etc.* This “top-down” synthesis of GQDs usually employ oxidative cleavage under harsh conditions, such as refluxing in a high concentration of oxidizing acid (*e.g.* HNO_3_) or electrochemical oxidation at high potential (usually larger than 10 V). However, the obtained GQDs usually have a high oxidation state, leading to cathodic ECL performance at extremely low voltages. Direct preparation of GQDs with anodic ECL remains great challenges. It has been proven that hydrazide-modified GQDs (HM-GQDs) synthesized through post modification of acid cleaved GQDs, have anodic ECL at a low potential (∼0.4 V *vs.* Ag/AgCl) because of the introduced luminol-like units.^45,46^

Inspired by these researches, luminol is chosen in this work as the precursor for one-step synthesis of nitrogen doped graphene quantum dots (NGQDs) with anodic ECL. As known, luminol (3-amino-phthalohydrazide) is one of the most commonly used chemiluminescent reagents, which also has anodic ECL properties due to the presence of hydrazide groups. As shown in Fig. 1, NGQDs were directly synthesized through molecular fusion of luminol in one-step hydrothermal reaction.

Fig. S1A (ESI†) shows the fluorescence intensities of the obtained materials synthesized at different reaction temperatures. When the synthesis temperature increases, the fluorescence intensity of the product significantly increases, indicating high efficiency for the synthesis of GQDs at high temperature resulting from improved degree of fusion between precursor molecules. As the withstand temperature of the used Teflon-lined autoclave is 220 °C, synthesis of NGQDs at 200 °C is selected for further investigation from the point of safety. The effect of reaction time on the fluorescence intensity of the obtained product was further optimized. As revealed in Fig. S1B (ESI†), increasing the synthesis time can improve the fluorescence intensity when the synthesis time is short. The highest fluorescence intensity of the product was obtained at reaction for 10 h. The further increase of the reaction time leads to the decrease of the fluorescence intensity instead. In addition, some solid precipitation was observed at the bottom of the resulting solution when the synthesis time was 12 h, proving the generating of some large particle. Thus, NGQDs was synthesized at 10 h in the subsequent experiments.

In addition to the synthesized GQDs, the solution obtained in hydrothermal synthesis commonly contains unreacted precursors or medium molecules. Dialysis is a convenient method to remove these unreacted small molecules. As shown in Fig. S2A (ESI†), when the product was dialyzed in ultrapure water for 4 h, the surrounding solution outside the dialysis bag has significant ultraviolet (UV) absorption with a maximum absorption wavelength at 348 nm. The effect of dialysis time on the absorbance at 348 nm was further investigated (Fig. S2B in ESI†). As seen, the absorbance of the solution significantly decreased with the increase of dialysis time. After 48 h of dialysis, the absorbance of the solution was almost zero, proving the effective removal of the unreacted small molecules. Thus, dialysis for 48 h was chosen in the preparation of NGQDs. When calculated based on the used precursor, the synthesis yield is 47%. Thus, the proposed one-step hydro-thermal synthesis has the advantages of simple method, convenient operation and high yield. Compared with ECL using luminol in solution, solid NGQD nanoemitter can be confined in solid materials (*e.g.* porous nanomaterials, film, gel) and has great potential in the fields of solid luminescence, visual detection, resonance energy transfer, *etc.*

### 3.2 Characterization of NGQDs

**Fig. 2:**
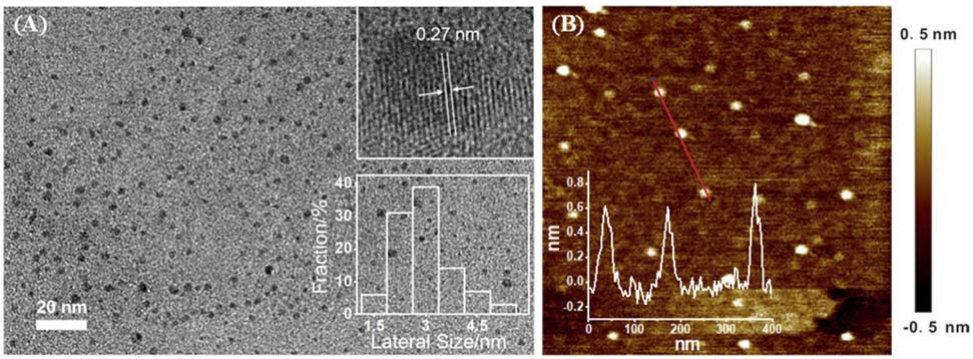
(A) TEM images of NGQDs. Top inset is high-resolution TEM (HRTEM) image with indicated lattice. Top inset is the size distribution of NGQDs. (B) AFM image of NGQDs. Inset is the corresponding height profile along the indicated line.

The size of NGQDs was characterized by transmission electron microscopy (TEM). Fig. 2A displays TEM images of NGQDs at different magnification. As seen, no aggregation of NGQDs is found, indicating good dispersion ability. The high-resolution TEM image (HRTEM) shows good crystallinity of NGQDs (top inset of Fig. 2A). Owing to the introduction of N atoms form the precursor, the lattice spacing of 0.27 nm is slightly larger than the (1120) lattice fringes of graphene. The size of NGQDs ranges from 1.5 nm to 5 nm with an average size of 3 nm (bottom inset of Fig. 2A). Thus, NGQDs have ultrasmall and relatively uniform size. The thickness of NGQDs was characterized by atomic force microscope (AFM). As shown in Fig. 2B, NGQDs have the thickness of ∼0.6 nm, indicating singly-layered graphene structure. In the XRD pattern, NGQDs exhibits a broad interlayer peak, indicating graphite (002) diffraction peak (Fig. S3 in ESI†).^47^

**Fig. 3:**
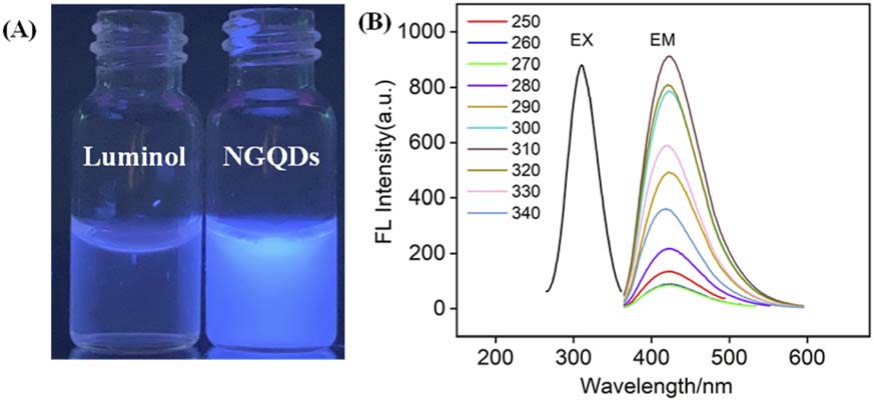
(A) Digital images of luminol or NGQD solution illuminated by 365 nm UV light. (B) Fluorescence excitation (EX) and emission (EM) spectra of NGQDs. The EM spectrum is obtained with excitation wavelength ranging from 250 to 340 nm. The increment in excitation wavelength between two adjacent spectra is 10 nm.

Owning to the ultrasmall size of NGQDs, the energy band is opened, resulting in the fluorescence. As shown in Fig. 3A, NGQDs have strong blue fluorescence com-pared with the precursor, luminol solution. Fig. 3B demonstrates the fluorescence excitation and emission spectra of NGQDs. The maximum excitation and emission wavelengths are located at 300 nm and 420 nm, respectively. When NGQD solution is irradiated with different wavelengths of emission light (250–340 nm), the maximum emission wavelength of NGQDs does not change. Thus, NGQDs have an excitation-independent fluorescence, indicating the uniform surface structure.^28^ Therefore, even when the excitation wavelength changes, the fluorescence color and emission peak of NGQDs do not change. These characteristics might be beneficial for making fluorescent labels or imaging, *etc.*^28,48^

To verify the difference between NGQDs and the employed precursors, fluorescence excitation and emission spectra of luminol were given in Fig. S4 (ESI†). As seen, luminol has a maximum excitation spectrum of ∼425 nm and an excitation-dependent fluorescence emission. These characteristics are significantly different from that of NGQDs, proving different structures and properties between NGQDs and the precursor. The absolute photoluminescence quantum yield of the NGQDs was measured to be 13.2%.

**Fig. 4:**
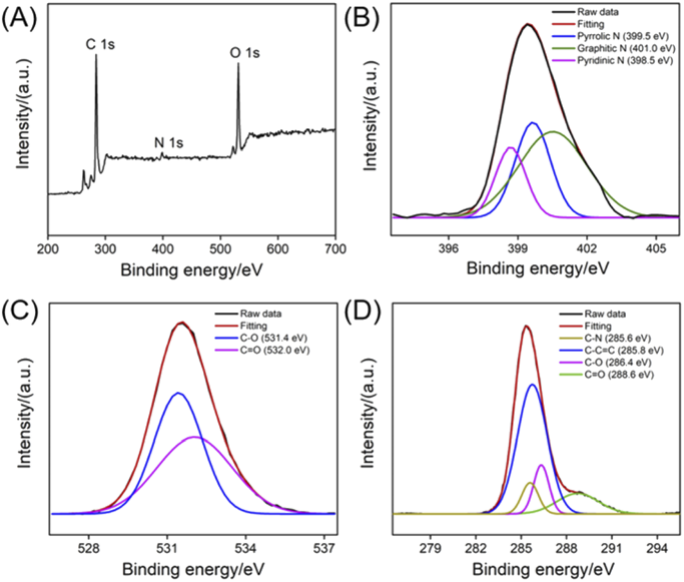
XPS survey spectrum (A) and high-resolution C 1s (B), O 1s (C), or N 1s (D) spectra of NGQDs.

The chemical composition of NGQDs is investigated using X-ray photoelectron spectroscopy (XPS). As shown in Fig. 4, the XPS survey spectrum reveals three elements including C, O, and N with atomic percentages of 69.3%, 3.5% and 27.2%, respectively, indicating abundant N and oxygenated groups (Fig. 4A). As shown in the high-resolution C 1s spectrum, NGQDs contain sp^2^ carbon (C–C=C), C–O, C=O and C–N groups (Fig. 4B). The high-resolution O 1s spectrum reveals two peaks corresponding to C=O and C–O groups (Fig. 4C). As shown in the high-resolution N 1s spectrum, peaks corresponding quaternary N (graphite N), pyridinic N pyrrole N are found, suggesting N doping in GQDs (Fig. 4D). Thus, NGQDs contains the functional hydrazide groups. The elemental analysis of NGQDs was also performed. The N/C ratio in elemental analysis (0.292) is slightly higher that of XPS measurement (0.219). This might be ascribed the fact that XPS can only provide surface information, and oxygen adsorption might occur on the surface of NGQDs, resulting in a low measured N/C ratio.

The UV-vis absorption spectrum of the as-prepared NGQDs is given in Fig. S5A.† As shown, NGQDs have obvious absorption in the wavelength range from 250 nm to 450 nm. The corresponding optical band gap (*E*_g_) could be estimated using Tauc plot.^49–51^ Briefly, it is the curve of (*αhν*)^*r*^*versus hν* converted from the UV-vis spectrum, where *α*, *h*, and *ν* are the absorption coefficient, Planck constant, and light frequency, respectively (Fig. S5B†). The *E*_g_ value of NGQDs was determined to be 4.87 eV by measuring the *x*-axis intercept of an extrapolated line from the linear regime of the curve (Fig. S5B,† black dotted line).

High stability is critical for the practical use of NGQDs. Thus, the stability of NGQDs is investigated by measuring the fluorescence intensity under continuous exposure (3 h) to UV light irradiation (365 nm, 20 W), long-term storage (30 days) at indoor environment, and in presence of salt (NaCl, 0.5 M). The fluorescence intensity of NGQDs remains 97.9%, 99.4% and 99.0% of the original signals, respectively, indicating high stability under these three harsh conditions.

### 3.3 ECL properties of NGQDs in presence of catechol

Electrochemiluminescence combines the advantages of electrochemistry and chemiluminescence, which makes it advantageous in sensitivity and selectivity. In addition to traditional ECL emitters, such as ruthenium bipyridine, some nanomaterials, including CdTe QDs, ZnS QDs, gold or silver nanoclusters and other composite nanomaterials, have been developed as ECL emitters. Amongst, carbonaceous nanomaterials have attracted much attention because of their good biocompatibility and adjustable ECL properties.

**Fig. 5:**
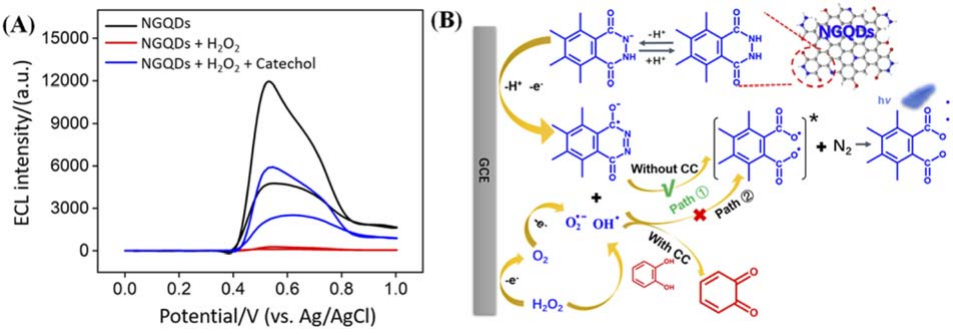
(A) ECL intensity–potential curves of NGQDs (100 μg mL^−1^) in absence or presence of H_2_O_2_ (1 mM) or the mixture of H_2_O_2_ (1 mM) and catechol (7 μM) in PBS (0.1 M, pH = 9.0). (B) The possible ECL mechanisms of NGQDs in presence of H_2_O_2_ (path ①). Path ② is the possible mechanism of the decrease of ECL intensity with CC.

Fig. 5A shows ECL intensity–potential curves of NGQDs in absence or presence of H_2_O_2_ in PBS (0.1 M, pH = 9.0). The introduction of H_2_O_2_ lead to significantly increased ECL signal of NGQDs. The possible ECL mechanism is demonstrated in Fig. 5B. During scanning from anionic to anodic potentials at GCE, the abundant hydrazide groups on NGQDs are firstly oxidized at the electrode to form anion. Then the anion reacts with reactive oxidative species (ROS) that are electrochemically generated from H_2_O_2_ to produce the excited-state anion, which finally emits light (Fig. 5B, path ①). Thus, ECL of NGQDs is facilitated in the presence of H_2_O_2_, due to the ability in producing ROS. When catechol is added, the ECL intensity significantly decreased. As an antioxidant, catechol might inhibit luminescence by competing with luminol for ROS and consuming some oxygen species (Fig. 5B, path ②). To prove the possible mechanism, the ECL intensity of NGQDs in presence of resorcinol, an isomer of catechol, is investigated. It was found that resorcinol cannot decrease the ECL intensity of NGQDs. This might be ascribed that resorcinol cannot be oxidized to form quinone structure, leading to no change of ROS in the system.

### 3.4 Electrochemiluminescence determination of catechol

**Fig. 6:**
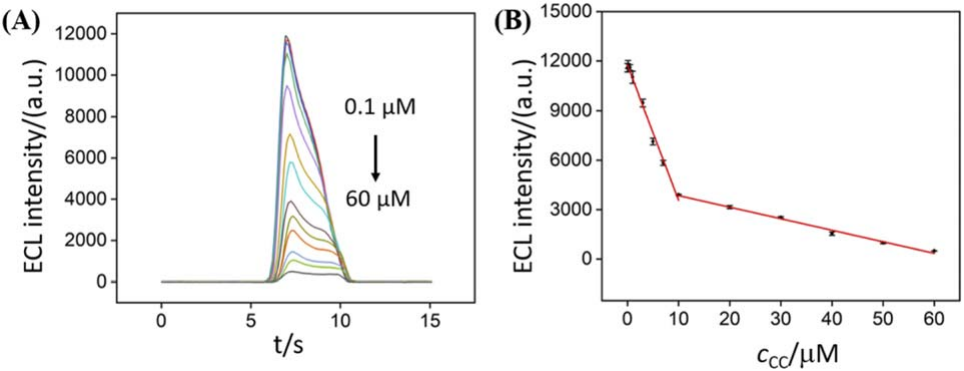
(A) ECL intensity of NGQDs in response to different concentrations of CC. (B) Calibration curve for the ECL determination of CC.

ECL determination of catechol was carried out based on the quenching of ECL signal of NGQDs by catechol. As shown in Fig. 6, the ECL signal of NGQDs significantly decreases when different concentrations of catechol are added. The ECL intensity has a linear relationship with the concentration of catechol ranged from 100 nM to 10 μM and 10 μM to 60 μM. The limit of detection (LOD) is 42 nM at a signal-to-noise ratio of 3 (S/N = 3). The LOD is lower than that obtained on graphene/multiwall carbon nano-tube/gold nanocluster (GP/MWCNTs/AuNCs),^52^ Al-doped silica modified carbon paste electrode (Al–SiO_2_/CPE),^53^ carbon nanocages/reduced graphene oxide modified GCE (CNCs–rGO/GCE),^54^ Au–Pd nanoflower/reduced graphene oxide modified GCE (Au–Pd NF/rGO/GCE),^55^ but is lower than that obtained on carbon dots/copper-based metal organic framework modified GCE (CDs@HKUST-1/GCE).^3^ In comparison with other sensing strategy, our developed ECL sensor has advantages of simple synthesis, convenient operation, and sensitivity detection.

### 3.5 Determination of catechol in environmental water

The ECL sensing based on NGQD emitter is applied for the determination of CC in lake water using standard addition method. As shown in Table 1, the recoveries ranges from 99.4% to 104.0% with a low relative standard deviation (RSD), indicating good reliability.

**Table 1:** ECL determination of CC in lake water using the developed NGQD emitter

Sample^a^	Added (μM)	Found (μM)	RSD (%, *n* = 3)	Recovery (%)
Lake water^a^	20.0	20.8	1.6	104.0
	50.0	50.5	2.5	101.0
	100.0	99.4	1.5	99.4

a The samples with added CC were diluted by a factor of 10. The added concentration of CC was the concentration before dilution.

## 4. Conclusions

In summary, we have developed an electrochemiluminescence sensing platform for sensitive ECL determination of catechol based NGQD emitter. NGQDs are synthesized through one-step hydrothermal process using molecular fusion of luminol. The proposed synthesis protocol has advantages of simple method, convenient operation, and high yield. NGQDs exhibit anodic ECL with high signal in presence of H_2_O_2_ owing to the existed hydrazide groups. Sensitive detection of catechol is realized because it can decrease ECL signal of NGQDs by competing with luminol for reactive oxygen group and consuming some oxygen species. Compared with solution-based ECL probe, the developed nanocarbon luminescent probe of NGQDs has potential in solid-state light-emitting devices, composite materials and other fields.

## Conflicts of interest

The authors declare no conflict of interest.

## Supplementary Material

RA-012-D2RA04209F-s001
