# Supplementary material for: Facile synthesis of nitrogen-doped graphene quantum dots as nanocarbon emitters for sensitive detection of catechol
Source: RSC Adv. 2022 Sep 9;12(39):25778–85. doi: 10.1039/d2ra04209f (PMC9461467; doi:10.1039/d2ra04209f)
Supplement: RA-012-D2RA04209F-s001 [file RA-012-D2RA04209F-s001.pdf]

Electronic Supplementary Material (ESI) for RSC Advances.
This journal is © The Royal Society of Chemistry 2022

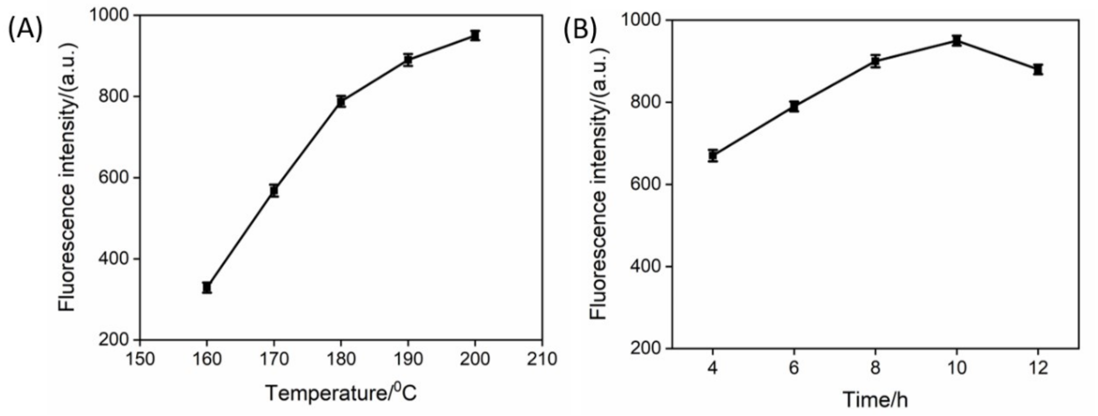

Fig. S1 (A) Fluorescence intensity of product (100 μg/mL) synthesized with different temperature (A) or reaction time (B).

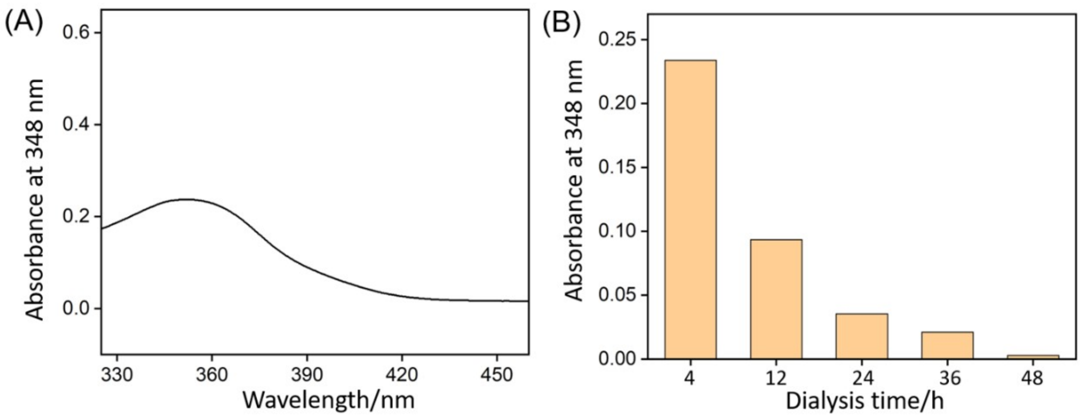

Fig. S2 (A) UV-Vis spectrum of the surrounding solution when the product obtained in hydrothermal synthesis was dialyzed for 4h. (B) The absorbance at 348 nm of the surrounding solutions obtained in different dialysis time.

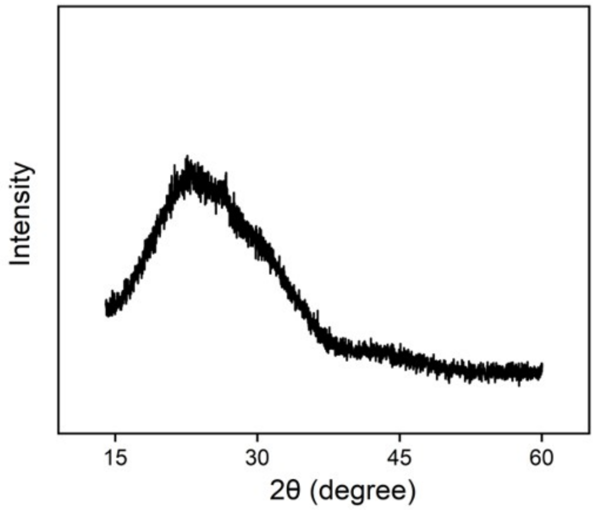

Fig. S3 XRD patterns of NGQDs.

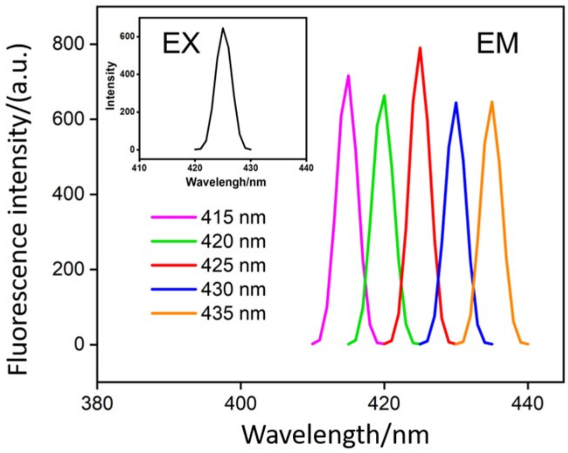

Fig. S4 Fluorescence excitation (EX) and emission (EM) spectra of luminol (5mM). The emission spectra were obtained at different excitation wavelength as indicated in the figure.

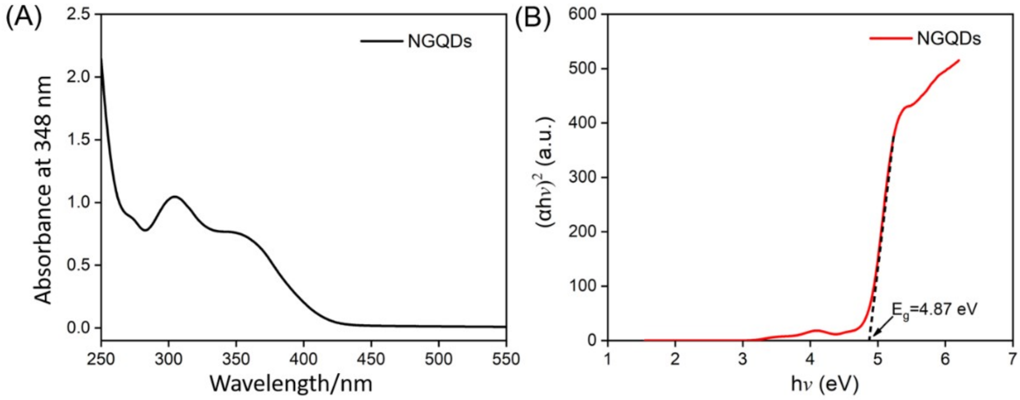

Fig. S5 (A) UV-Vis spectrum and (B) Tauc plot of the NGQDs.
